# A Case in Which HLA-DR4 is Involved in the Development of Complex Immune-Related Endocrinological Adverse Events following Combination Therapy with Nivolumab and Ipilimumab

**DOI:** 10.1155/2024/4662803

**Published:** 2024-03-22

**Authors:** Yonekazu Kidawara, Manabu Kadoya, Miki Kakutani-Hatayama, Hidenori Koyama

**Affiliations:** Department of Diabetes, Endocrinology and Clinical Immunology, School of Medicine, Hyogo Medical University, Nishinomiya, Hyogo, Japan

## Abstract

Immune checkpoint inhibitors (ICIs) have become a focal point in cancer immunotherapy, though their utilization is also linked to the occurrence of diverse immune-related adverse events (irAEs). Herein, we present details of a 42-year-old woman diagnosed with a malignant vaginal melanoma who underwent ICI therapy with the combination of nivolumab and ipilimumab. Approximately two months after initiating therapy, the patient manifested destructive thyroiditis and fulminant type 1 diabetes mellitus, thus necessitating intensive insulin therapy. Following the onset of adrenocorticotropic hormone deficiency, frequent hypoglycemic episodes prompted the initiation of replacement therapy with hydrocortisone. Human leukocyte antigen (HLA)-DNA typing revealed the presence of HLA-DRB1^*∗*^04 : 05 and DQB1^*∗*^04 : 01. HLA-DR4 has been suggested to be associated with the development of multiple endocrine irAEs. This is the first reported case of three endocrine irAEs occurring within a short period, in which the presence of HLA-DR4 may have contributed to the pathogenesis.

## 1. Introduction

Malignant neoplasm is one of the leading causes of morbidity and mortality and is difficult to treat in many affected patients [1]. The introduction of immune checkpoint inhibitors (ICIs) has provided revolutionary advances in the field of cancer therapy by providing significant improvements in clinical outcomes, such as prolonged long-term survival and sustained tumor shrinkage, for patients affected by a variety of cancer types [2, 3]. Monoclonal antibodies, such as anti-programmed cell death 1 (anti-PD-1) (nivolumab, pembrolizumab, and cemiplimab) and anti-cytotoxic T-lymphocyte antigen-4 (anti-CTLA-4) (ipilimumab and tremelimumab), activate T cells and have been shown to exhibit efficacy against a wide spectrum of malignant tumors, leading to the use of ICIs as one of the most promising therapy options presently available. However, ICI administration has been shown to be associated with immune-related adverse events (irAEs), which can have effects on all organs throughout the body, and is potentially severe or even fatal. Therefore, irAEs pose a significant challenge in the current landscape of cancer immunotherapy [4]. Notably, irAEs are typically constituted of endocrine disorders, with the mechanism speculated to involve activation of autoimmune responses targeting endocrine organs, leading to hypophysitis, thyroiditis, and/or fulminant type 1 diabetes mellitus [5], though it is rare for all three conditions to occur within a short period. Interestingly, human leukocyte antigen (HLA) was recently shown to be a possible predictor of endocrine irAEs [6]. Presented here is the first reported case in which HLA-DR4 may have been associated with the development of three endocrine irAEs observed within a short period.

## 2. Case Presentation

A 42-year-old woman was diagnosed with a malignant melanoma in the vaginal area and treated with nivolumab and ipilimumab combination therapy. There were no coexisting conditions nor regular medication history, and the patient had been in a healthy state prior to the diagnosis. Laboratory assessments performed during the seven-week period following the commencement of treatment indicated the presence of thyrotoxicosis, characterized by a thyroid-stimulating hormone (TSH) level of 0.021 mIU/L (0.5–5.0 mIU/L) and a free thyroxine (FT4) level of 44.9 pmol/L (11.6–21.9 pmol/L). After 10 weeks, the emergence of increased thirst prompted additional laboratory examinations, which showed an elevated level of hemoglobin A1c (HbA1c) at 6.7% (4.6–6.2%); thus, the patient was referred to the Department of Diabetes, Endocrinology, and Metabolism.

**Figure 1:**
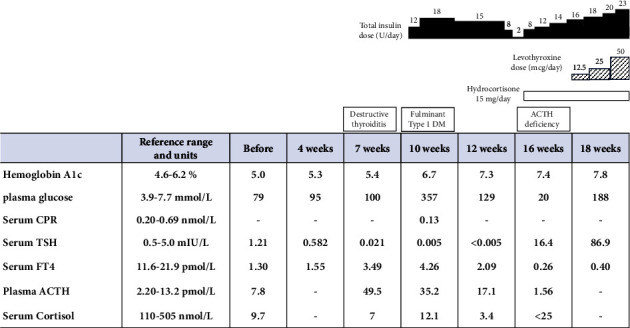
Time course for clinical parameters, diagnosis, and treatment. Nivolumab and ipilimumab were administered at 0, 4, 7, 10, 12, 16, and 18 weeks. CPR: C-peptide reactivity; ACTH: adrenocorticotropic hormone; FT4: free thyroxine; TSH: thyroid-stimulating hormone.

Changes in serum or plasma concentrations of HbA1c, TSH, FT4, adrenocorticotrophic hormone (ACTH), and cortisol during the clinical course of the disease are presented in Figure 1. The patient's height was 149 cm, and weight was 40.1 kg. There were no discernible physical manifestations indicative of thyrotoxicosis, such as hand tremors, though she did show weight loss of approximately 2 kg over the course of one month, along with palpitations. Blood pressure was 96/53 mmHg, pulse 83 beats per minute, and body temperature 36.7°C. For assessment of thyroid function, the initial laboratory examinations upon admission indicated thyrotoxicosis, with TSH and FT4 levels at 0.005 mIU/L (0.5–5.0 mIU/L) and 54.8 pmol/L (11.6–21.9 pmol/L), respectively. All thyroid-related autoantibodies showed mild elevation, with the level of thyroid-stimulating antibody (TSAb) as 218% (<110%), thyrotropin receptor antibody (TRAb) as 2.9 IU/L (<2 IU/L), anti-thyroid peroxidase (anti-TPO) antibody as 9.8 IU/mL (<3.3 IU/mL), and anti-thyroglobulin (anti-Tg) antibody 29.7 IU/mL (<19.3 IU/mL). No evidence of thyroid enlargement or elevated blood flow was observed in thyroid ultrasonography findings. On the other hand, plasma glucose level was 19.8 mmol/L (3.9–7.7 mmol/L), indicating abnormal glucose metabolism. The level of antiglutamic acid decarboxylase antibodies and islet autoantibodies was below 5 U/mL (5 U/mL), with insulin secretory capacity also low, and blood C-peptide reactivity (CPR) two hours after a meal at 0.13 nmol/L (0.20–0.69 nmol/L) and urinary CPR at 4.8 mcg/day (29.2–167 mcg/day), along with an elevated blood ketone level of 2028 mcmol/L (0–130 mcmol/L). As for family history, the sister of the patient had type 1 diabetes mellitus. A diagnosis of fulminant type 1 diabetes mellitus was determined, and based on the clinical course and endocrine findings, the patient was determined to have thyroiditis and fulminant type 1 diabetes mellitus with irAEs due to the combination therapy with nivolumab and ipilimumab. At that time, there were no decreases in blood ACTH or cortisol levels noted, and adrenal function appeared to be normal.

**Figure 2:**
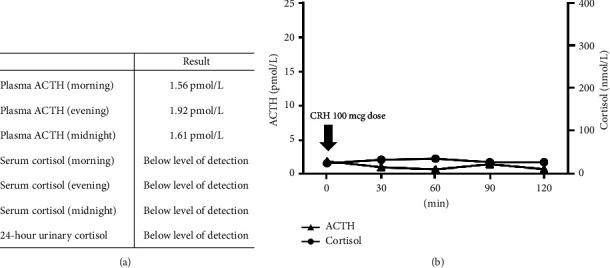
(a) Diurnal variations for blood ACTH and cortisol, and urinary cortisol indicated low levels. (b) Corticotropin-releasing hormone (CRH) stimulation testing showed no change in adrenocorticotropic hormone (ACTH) or cortisol level. The CRH stimulation test was conducted in the morning under a fasting condition.

**Figure 3:**
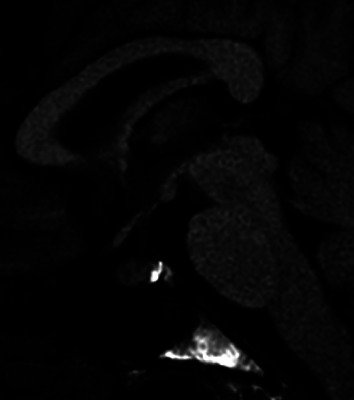
Pituitary contrast-enhanced MRI revealed heterogeneous enhancement of the pituitary gland.

Subsequently, thyrotoxicity was found to be spontaneously resolved and insulin therapy was initiated to correct glucose metabolism. For a short period, the patient remained free from hypoglycemia. However, at 16 weeks following commencement of treatment, sudden and recurrent episodes of hypoglycemia were noted, accompanied by subjective manifestations such as fatigue and reduced appetite. Blood pressure was 90/48 mmHg, pulse 88 beats per minute, and body temperature 35.8°C. Laboratory examinations revealed that despite a low fasting blood glucose level of 1.1 mmol/L (3.9–6.0 mmol/L), ACTH was 1.56 pmol/L (2.20–13.2 pmol/L), serum cortisol was <25 nmol/L (110–505 nmol/L), and dehydroepiandrosterone sulfate (DHEA-S) was 0.70 mcmol/L (0.51–6.26), each low. In addition, a low serum sodium level of 133 mmol/L (138–145 mmol/L) was noted, suggesting the possibility of secondary hypoadrenocorticism. There were diurnal variations in ACTH/cortisol in low levels as well as in low urinary cortisol levels, while a corticotropin-releasing hormone (CRH) stimulation test demonstrated no significant response for either ACTH or cortisol level (Figure 2). A pituitary contrast-enhanced MRI (Figure 3) revealed heterogeneous enhancement of the pituitary gland, which is a characteristic finding of the ICI-induced hypophysitis.

Based on these findings, the patient was diagnosed with ACTH deficiency associated with hypophysitis due to irAEs, and hydrocortisone treatment was started at 15 mg/day. Subsequently, due to an increasing blood glucose level, intensified insulin therapy was reinstated, while thyroid function was concurrently decreased, with a TSH level of 16.4 mIU/L (0.5–5.0 mIU/L) and FT4 level of 0.03 mIU/L (0.5–5.0 mIU/L). Thus, approximately one week after commencing hydrocortisone replacement therapy, levothyroxine replacement therapy was initiated at a low dose and gradually escalated. Ultimately, the patient was discharged without experiencing hypoglycemia and continued to receive hydrocortisone at 15 mg/day, levothyroxine at 50 mcg/day, and insulin therapy.

To more closely examine the concurrent manifestation of three endocrine irAEs in this case, HLA testing was conducted during the follow-up course. The methodology employed included HLA-DNA typing, utilization of blood samples, and a polymerase chain reaction-sequence-based typing method. Those results revealed the presence of HLA-DRB1^*∗*^04 : 05 (HLA-DR4) and DQB1^*∗*^04 : 01.

## 3. Discussion

Presented here are findings from the first reported case affected by three endocrine irAEs, hypophysitis, thyroiditis, and fulminant type 1 diabetes mellitus, within a short period. Moreover, these multiple endocrine disorders were associated with HLA-DR4 recognition. An association between autoimmune polyendocrine syndrome (APS) and HLA type in the context of irAEs has recently been speculated. APS is characterized by the simultaneous presence of multiple autoimmune conditions, including the three types recognized to be associated with HLA-DR4. HLA is a genetic background factor associated with autoimmune disease that has a crucial role in developing immune response, with its genetic polymorphism closely linked to individual variations in intensity of immune response to foreign antigens. Notably, the presence of HLA-DR4 is recognized as indicative of susceptibility to type 1 diabetes mellitus and is also associated with autoimmune thyroid diseases, which are encompassed within APS.

In addition to the present case, other studies have documented the development of multiple endocrine disorders associated with irAEs following treatment with ICIs, with HLA typing performed in 15 reported cases [7–17] (Table 1). Age, gender, country, and tumor types in all of those cases have been verified, with PD-1 or programmed death-ligand 1 (PD-L1) inhibitors administered in each. All of the affected patients exhibited type 1 diabetes mellitus, while 14 were diagnosed with thyroid disease. Among those as well as the present case, four had three endocrine disorders, three were diagnosed with hypopituitarism, and two were presented with Addison's disease. Concerning HLA type, HLA-DR4 was the most prevalent in 11 cases and the predominant type in three of the four cases with three endocrine disorders. These findings show that the presence of HLA-DR4 is important for predicting susceptibility to development of three endocrine irAEs. Furthermore, a previous study reported that the percentage of spontaneous APS type 2 patients with HLA-DR4 was 35.2%, while that of patients with ICI-induced APS type 2 was notably higher at 71.4% [6]. Together, these results suggest that the correlation of multiple endocrine disorders with irAEs and HLA-DR4 may be more pronounced than previously expected.

As noted previously, the presence of HLA-DR4 is recognized to be associated with type 1 diabetes and autoimmune thyroid disease, though reports are limited regarding its association with hypophysitis. A study conducted in Japan that compared the frequency of HLA-DR4 in patients with isolated ACTH deficiency (IAD) to that in healthy controls found a significant association in patients with idiopathic IAD, while no such association was found in patients with PD-1 antibody-induced IAD [18]. It is important to note that the reported prevalence of PD-1 antibody-induced IAD itself is low, though the number of patients in the study mentioned earlier was small, totaling only 10 cases. Notably, HLA-DR4 was present in two of the three cases with three endocrine immune-related adverse events including hypophysitis, which is the same as noted in the present patient.

The rate of incidence of pituitary damage caused by ICIs has been reported to range from 0.3% to 1.1% for anti-PD-1/anti-PD-L1 antibodies, while that was found to be 1.8% to 5.6% for anti-CTLA-4 antibodies, with even higher rates of 7.68% to 10.5% were noted for cases with combination therapy using both anti-CTLA-4 and anti-PD-1 antibodies [19]. Although no reports suggesting an association between HLA-DR4 and the development of pituitary inflammation induced by anti-CTLA-4 antibodies have been presented, it is plausible that the combination therapy with anti-PD-1 and anti-CTLA-4 antibodies contributed to the development of hypophysitis in the present case, regardless of the presence of HLA-DR4. Nevertheless, it should also be noted that HLA-DR4, known for its strong association with the development of type 1 diabetes and autoimmune thyroiditis, might contribute to an elevated risk of experiencing three endocrine immune-related adverse events, even though its presence may not be directly linked to hypophysitis development.

In conclusion, treatment with nivolumab and ipilimumab combination therapy resulted in the emergence of three endocrine irAEs in the present patient, with HLA-DR4 considered to have possibly contributed to the pathogenesis. The notable likelihood of HLA-DR4 involvement in the development of three endocrine irAEs underscores the utility of HLA typing for assessing the risk of ectopic and asynchronous onset of irAEs. Nevertheless, the appearance of multiple irAEs within a short period is rare and the limited number of cases hinders the establishment of a conclusive association with HLA type. Therefore, HLA typing is not currently recommended as a screening test for determining the onset of irAEs and it will be essential to accumulate additional data from future studies to further elucidate this potential association. Among irAEs, type 1 diabetes mellitus and hypoadrenocorticism are known to be life-threatening conditions if not appropriately treated. If it is proven to be a valuable tool for assessing the risk associated with endocrine irAEs, HLA-type screening may facilitate early diagnosis and treatment of affected patients, and potentially serve as an important method for managing related adverse events.

## Figures and Tables

**Table 1:** Reported cases.

Patient no	Age (years)	Gender	Country	Tumor type	Drug (ICIs)	T1DM time of onset	Thyroid disease's time of onset	Hypophysitis time of onset	Addison's disease time of onset	HLA type	Reference no
1	54	Male	USA	Melanoma	Nivolumab ipilimumab	20 weeks	2 weeks	20 weeks	—	A2, DQB1^*∗*^06 : 02	[7]
2	78	Female	Italy	Melanoma	Pembrolizumab	4 weeks	10 weeks	—	10 weeks	DRB1^*∗*^04 : 16, DQB1^*∗*^02 : 05	[8]
3	60	Male	Italy	Lung adenocarcinoma	Atezolizumab	6 weeks	—	12 weeks	12 weeks	DRB1^*∗*^04, DQB1^*∗*^03	[9]
4	68	Female	Japan	Renal cell carcinoma	Nivolumab	13 weeks	2 weeks	—	—	DRB1^*∗*^09 : 01, DQB1^*∗*^03 : 03	[10]
5	66	Female	USA	Sarcomatoid SCC	PD-1 inhibitor	7 weeks	N/A	—	—	DR3-DQ2, DR4-DQ8	[11]
6	55	Female	USA	Melanoma	Nivolumab, ipilimumab	5 months	N/A	—	—	A2, DRB1^*∗*^04	[12]
7	64	Female	USA	Melanoma	Pembrolizumab	<1 month	N/A	—	—	DRB1^*∗*^04	[12]
8	68	Male	Korea	SCC	Pembrolizumab	7 weeks	N/A	—	—	DRB1^*∗*^09 : 01, DQB1^*∗*^03 : 03	[13]
9	48	Male	Japan	Parotid gland adenocarcinoma	Nivolumab	17 weeks	15 weeks	—	—	DRB1^*∗*^04 : 05	[14]
10	61	Male	Belgium	NSCLC	Pembrolizumab	8 weeks	8 weeks	—	—	DRB1^*∗*^04, DQA1^*∗*^03 : 01, DQB1^*∗*^03 : 02	[15]
11	82	Male	Australia	SCC	Pembrolizumab	9 weeks	N/A	—	—	DRB1^*∗*^04, DQB1^*∗*^03 : 02	[16]
12	23	Male	Australia	Melanoma	Pembrolizumab	8 weeks	N/A	—	—	DRB1^*∗*^03,DRB1^*∗*^04 DQB1^*∗*^03 : 02	[16]
13	83	Male	Belgium	Melanoma	Pembrolizumab	N/A	N/A	—	—	DRB1^*∗*^01 : 01, DQA1^*∗*^01, DQB1^*∗*^05 : 01/DRB1^*∗*^16 : 01, DQA1^*∗*^01, DQB1^*∗*^05 : 02	[17]
14	65	Male	Belgium	Melanoma	Pembrolizumab	N/A	N/A	—	—	DRB1^*∗*^04 : 01, DQA1^*∗*^02, DQB1^*∗*^02 : 02/DRB1^*∗*^07 : 01, DQA1^*∗*^03, DQB1^*∗*^03 : 01	[17]
Present case	42	Female	Japan	Melanoma	Nivolumab, ipilimumab	10 weeks	7 weeks	16 weeks	—	DRB1^*∗*^04 : 05, DQB1^*∗*^04 : 01	—

ICIs, immune checkpoint inhibitors; NSCLC, non-small cell lung cancer; SCC, squamous cell carcinoma; T1DM, type 1 diabetes mellitus; N/A, not available.

## Data Availability

The data used to support the findings of the study are included within the article.
